# Paediatric autoimmune and autoinflammatory conditions associated with uveitis

**DOI:** 10.1177/2515841420966451

**Published:** 2020-11-02

**Authors:** Najiha Rahman, Harry Petrushkin, Ameenat Lola Solebo

**Affiliations:** Moorfields Eye Hospital NHS Foundation Trust, London, UK; National Institute for Health Research Biomedical Research Centre, Moorfields Eye Hospital NHS Foundation Trust and UCL Institute of Ophthalmology, London, UK; Moorfields Eye Hospital NHS Foundation Trust, London, UK; National Institute for Health Research Biomedical Research Centre, Moorfields Eye Hospital NHS Foundation Trust and UCL Institute of Ophthalmology, London, UK; Great Ormond Street Hospital for Children NHS Foundation Trust, London, UK; Population, Policy and Practice Programme, UCL Great Ormond Street Institute of Child Health, 30 Guilford Street, London WC1N 1EH, UK; National Institute for Health Research Biomedical Research Centre, Moorfields Eye Hospital NHS Foundation Trust and UCL Institute of Ophthalmology, London, UK; Great Ormond Street Hospital for Children NHS Foundation Trust, London, UK; National Institute for Health Research Biomedical Research Centre, UCL Great Ormond Street Institute of Child Health and Great Ormond Street Hospital, London, UK

**Keywords:** autoimmune, autoinflammation, child, uveitis

## Abstract

Childhood uveitis comprises a collection of heterogenous ocular phenotypes which are associated with a diverse range of childhood autoimmune and autoinflammatory disorders. Of these genetic and/or acquired disorders, juvenile idiopathic arthritis is the most common, affecting 30-80% of children with uveitis. Up to a third of children with uveitis have ‘isolated’ idiopathic disease and do not have an associated systemic disease which manifests in childhood. However, uveitis may be the presenting manifestation of disease; thus, the apparently well child who presents with uveitis may have isolated idiopathic disease, but they may have an evolving systemic disorder. The diagnosis of most of the associated disorders is reliant on clinical features rather than serological or genetic investigations, necessitating detailed medical history taking and systemic examination. Adequate control of inflammation is key to good visual outcomes, and multidisciplinary care is key to good broader health outcomes.

## Introduction

Uveitis, a descriptive term, encompasses a heterogeneous group of inflammatory eye disorders which are classified by anatomy (anterior, intermediate, posterior or ‘pan’uveitis; Table 1), by cause (e.g. infectious *versus* noninfectious, idiopathic *versus* known disorder) or by the occurrence of an associated systemic disorder [e.g. with juvenile idiopathic arthritis (JIA)].^1^

**Table 1.** Classification of uveitis based on SUN^a^ criteria (SUN).^1^

Type	Primary site of inflammation	Manifest conditions include
Anterior uveitis	Inflammation of the anterior chamber affecting the iris and anterior ciliary body	Iritis Iridocyclitis Anterior cyclitis
Intermediate uveitis	Inflammation of the vitreous	Pars planitis Posterior cyclitis Hyalitis
Posterior uveitis	Inflammation of the retina or choroid	Focal or diffuse choroiditis Chorioretinitis Retinochoroiditis Retinitis Neuroretinitis Retinal vasculitis
Panuveitis	Inflammation of the anterior chamber, vitreous and retina or choroid	

a The Standardization of Uveitis Nomenclature (SUN) group have provided a framework for uveitis research and clinical practice.

The uvea is the highly vascularized middle tract of the eye and the primary intraocular site for resident immune-competent cells. Infection, trauma or as-yet poorly understood inflammatory and immune-mediated processes can trigger abnormal responses leading to intraocular inflammation.

Childhood onset of uveitis is uncommon and typically chronic, relapsing or recurrent, with prolonged disease activity resulting in irreversible ocular structural damage.^2,3^ There is a paucity of robust evidence on the prevalence or incidence of paediatric uveitis. An often-cited UK annual incidence of 5 new cases per 100,000 children is derived from a 2003 retrospective observational study across three primary care centres with noncoincident ascertainment periods (1993–1994 at one centre, 1995 at another, 1996–2001 at the other).^2^ This incidence is similar to that reported by another European population-based study, which estimated the Finnish national annual incidence at 4/100,000, and the prevalence at 28/100,000 [95% confidence interval (CI), 17.1–38.6].^4^ Such a prevalence would suggest an approximate 3–6000 UK children living with uveitis. However, both these population-based studies on disease frequency are limited by their retrospective nature and incomplete national coverage.

Adult uveitis differs from childhood-onset disease in several ways. The most common paediatric disease manifestation (up to 90% of cases) is chronic anterior uveitis, which is less common in adult-onset disease.^5,6^ Children with uveitis exhibit a different spectrum of disease association than that seen in the adult population.^2,5,6^ Inflammatory sequelae such as glaucoma and cataract are more challenging to manage in children.^7–9^ There are also the additional obstacles of amblyopia, managing a chronic disease in a developing child and the difficulties of effective transition to adult care services.

## Immunopathogenesis of uveitis

The eye is thought to be an immune-privileged site, in that immune responses to apparently non-self-antigens are suppressed, preventing the collateral damage to ‘innocent bystander’ tissue that might occur in such a response. This offers protection for sites such as the brain, joint capsules and the eye, which have vital highly specialized roles in human function and limited capacity for regeneration.^10^ With immune privilege comes the absence of the full complement of cells and factors which have evolved to mitigate the inflammatory response. Consequently, breaches of the immune-privileged sites can overwhelm the resident protective systems, resulting in significant tissue damage.

In noninfectious uveitis, the cause of the breach of immune privilege is not yet understood, but what is clear is that it results in significant expansion of the intraocular expression of proinflammatory agents such as cytokines, chemokines and complement.^11^ The expression of these agents is mediated through either autoimmune (abnormal innate or immune response to self) or autoinflammatory (disordered adaptive or response to environmental signals) pathways.

Histopathological investigations have suggested different pathogenic pathways for childhood uveitis. Subsets of CD4+ and CD8+ T cells are thought to have either pathogenic (e.g. CD4+ Th17 cell lines) or protective (e.g. CD8+ Treg) roles, or in some cases both (Cd4+ γδT cells).^12^ Animal models of uveitis, particularly experimental autoimmune uveitis (EAU) in which a susceptible animal is immunized with retinal derived proteins, have provided valuable information on disease pathogenesis. This includes identifying tumour necrosis factor alpha (TNFα) as a therapeutic target, leading to the development of anti-TNFα monoclonal antibodies, such as adalimumab.^13^ No single animal model encapsulates the broad, heterogeneous spectrum of human disease. Plasma cells have been found to be the predominant cell type within ocular tissue samples of children with juvenile idiopathic arthritis–associated uveitis (JIAU). This finding suggests that the pathogenesis of JIAU may be antibody mediated and therefore more on the autoimmune, rather than autoinflammatory, end of the spectrum of diseases.^14^

## Autoimmune *versus* autoinflammatory disorders associated with childhood uveitis

**Figure 1.**
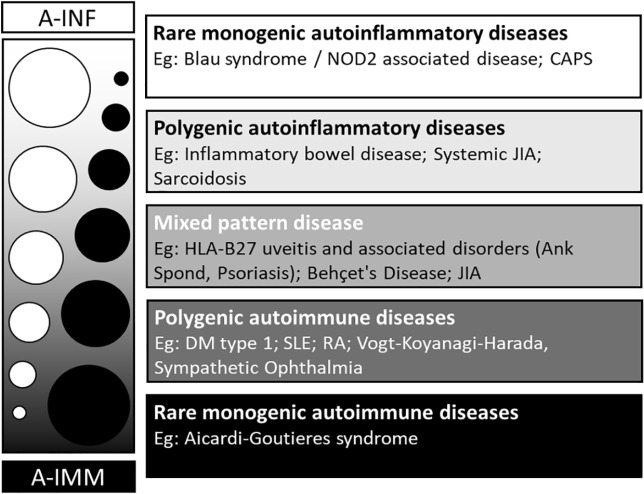
The spectrum of autoinflammation and autoimmunity. Source: Adapted from McGonagle and colleagues.^15^ A-IMM, autoimmunity; A-INF, autoinflammation; Ank Spond, ankylosing spondylitis; CAPS, cryopyrin-associated periodic syndromes; DM, diabetes mellitus; HLA, human leukocyte antigen; JIA, juvenile idiopathic arthritis; NOD2, nucleotide-binding oligomerization domain containing 2; RA, rheumatoid arthritis; SLE, systemic lupus erythematous.

In autoimmune disorders, there is self-directed inflammation, involving aberrant dendritic, B and T cells in response to native antigens.^15^ Pure autoimmune disease can become organ specific as aberrant major histocompatibility complex (MHC) expression and autoantibody formation develops over a number of years towards the target organ(s). In contrast, the self-directed inflammatory processes in autoinflammatory diseases are in response to so-called ‘danger’ signals which activate the innate immune cells, including macrophages, natural killer cells and neutrophils. These ‘danger’ signals may come in the form of non-self-antigens or tissue microdamage. Mutations in inflammasome-related genes have been strongly associated with autoinflammatory disease and may also be involved in autoimmune disorders.^16–20^ Inflammasomes are a group of powerful intracellular protein complexes which sense a wide range of inflammatory ‘danger’ signals and stimulate the release of the pro-inflammatory interleukin (IL) cytokines, IL-1β and IL-18. The best characterized members of the inflammasome complex are the nucleotide-binding and oligomerization domain (*Nod*)-like receptors (NLRs: NLRP1, NLRP3, NLRC4) and the absent in melanoma 2 (*AIM2*)-like receptors (ALRs). Rather than there being a distinct division between autoinflammatory and autoimmune disorders, immunological disease can be classified using a continuous ‘spectrum’ model which runs between the two (Figure 1). Uveitis is a well-recognized feature of autoinflammatory and mixed disorders, but is less commonly seen in predominantly autoimmune disorders (Table 2).

**Table 2.** 

Ocular manifestations of autoinflammatory and autoimmune disease.	Disease, % childhood uveitis cases^a^ (HLA/genetic association)	Systemic involvement	Common ocular phenotypes	Cited prevalence of uveitis
Monogenic autoinflammatory disease(s)	Blau syndrome, <1% (NOD2/CARD15)	Skin, joints	Panuveitis with multifocal chorioretinitis	78% (*n* = 38/50)^21^ Early-onset disease
	Cryopyrin-associated periodic syndromes, <0.1% (NLRP3/CIAS1)	Skin, joints, liver, deafness, meningitis	Anterior uveitis	55% (*n* = 17/31)^22^
Polygenic autoinflammatory disease(s)	Inflammatory bowel disease, <0.1% (NOD2/CARD15 HLA DRB1*1502, 0103)	Bowel, arthritis	Anterior uveitis	0.62–1.82% More prevalent in Crohn’s than ulcerative colitis^23^
	Sarcoid, 2–3% (HLA-DRB1*0301, *0401, DQB1*0301)	Skin, joints, lung (less common than in adults), liver	Chronic anterior uveitis, panuveitis	24–58%^24–26^
	TINU syndrome, <1% (HLA-DRB1*0102)	Kidney	Chronic anterior uveitis	<2% of all uveitis cases,^27^ median age of TINU onset is 15 years old^28^
Mixed pattern disease(s)	BD, 2–3% (HLA-B*5101, HLA-B*27)	Mucosa, skin, vasculitis	Panuveitis, retinal vasculitis	24–80% in childhood BD series^29–31^
	JIA, >60% (HLA DRB*0801, *1101, *1301, DPB1*02 (HLA-B*27, HLA-C*06, NOD/CARD15 in ERA and psoriatic)	Joints (seven distinct disorders)	Anterior uveitis	1–67%^32,33^ Dependent on JIA type^34–37^ Oligoarticular > enthesitis related, psoriatic, undifferentiated > polyarticular rheumatoid+ > polyarticular rheumatoid factor– > systemic
Polygenic autoimmune disease(s)	Vogt–Koyanagi–Harada syndrome, <1% (HLA-DRB1*04)	Skin, CNS	Panuveitis	0.5–16%, higher percentage in Saudi Arabia^38,39^
	Multiple sclerosis, <0.1% (HLA-DRB1*1501)	Central nervous system (CNS)	Optic neuritis, intermediate uveitis	Uveitis uncommon: 10–22% present with optic neuritis^40^
Monogenic autoimmune disease(s)	Aicardi Goutières syndrome, <0.1%	Skin, CNS, joints, lung	Glaucoma, episcleritis	Uveitis unknown^41^

BD, Behçet’s disease; CNS, central nervous system; ERA, enthesitis-related arthritis; HLA, human leukocyte antigen; JIA, juvenile idiopathic arthritis; TINU, tubulointerstitial nephritis and uveitis.

The commonest ‘cause’ of childhood uveitis is isolated idiopathic disease.

a Percentage of childhood uveitis cases in the US and Europe populations.^2,42^

**Figure 2.**
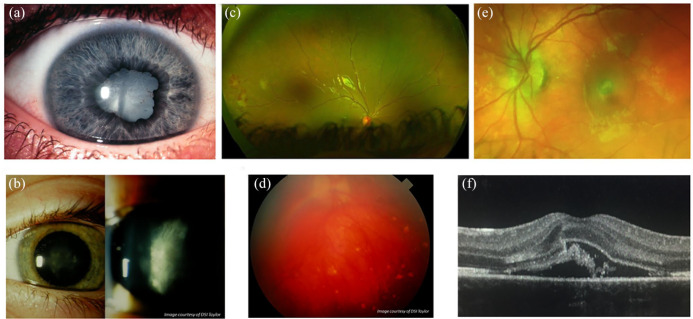
Manifestations of childhood uveitis. (a) Anterior uveitis: chronic anterior uveitis, with posterior synechiae and cataract in a child with delayed diagnosis of juvenile idiopathic arthritis–related uveitis. (b) Intermediate uveitis: pars planitis at the slit lamp in a child with idiopathic disease. (c) Posterior uveitis: Retinal vasculitis: periphlebitis, midperipheral neovascularization with evidence of retinal ischaemia in a child with sarcoidosis. (d) Panuveitis: vitritis, optic disc oedema and multifocal chorioretinitis in a child with idiopathic disease. (e) Sequelae of inflammation: retinal photograph of an inflammatory choroidal neovascular membrane (CNVM) in a child with idiopathic panuveitis. (f) Sequelae of inflammation: cross-sectional optical coherence tomography (OCT) and OCT angiography (en face choriocapillaris window) images of the same CNVM noted in (e). Images (a), (b) and (d) courtesy of DSI Taylor.

In childhood-onset uveitis, autoinflammatory and autoimmune conditions are more likely to present with certain manifestations of ocular inflammation (Table 2), but no single uveitic phenotype (Figure 2) is pathognomonic of a particular systemic inflammatory disease. For example, the majority of children with JIA develop chronic anterior uveitis (83%) with posterior synechiae, cataract and band keratopathy in uncontrolled disease, but panuveitis has also been noted in children with JIA.^43^

## Specific disorders associated with uveitis

Up to a third of children with uveitis have ‘isolated’ idiopathic disease and do not have an associated systemic disease which manifests in childhood. Other than JIA, which is itself an idiopathic disorder, this group is the largest ‘single’ group. Uveitis may be the presenting manifestation of disease; thus, the apparently well child who presents with uveitis may have isolated idiopathic disease, but they may have an evolving systemic disorder. We will now describe the autoimmune and autoinflammatory disorders associated with childhood uveitis, starting with the most prevalent disorders within paediatric uveitis clinics.

### Juvenile idiopathic arthritis

Juvenile idiopathic arthritis, the descriptive term for arthropathy of unknown cause which has lasted for more than 6 weeks, is the most common chronic inflammatory rheumatological disorder seen in children.^44^ In turn, JIA is the most common systemic disorder seen in paediatric uveitis clinics, affecting between 30% and 80% of children with uveitis. Within the ‘umbrella term’ of JIA sit seven distinct but overlapping subtypes, with differing incidences of uveitis (Table 2): systemic, oligoarticular (up to four joints affected), rheumatoid factor–positive (RF+) polyarticular (more than five joints affected), RF– polyarticular, enthesitis-related (ERA), psoriatic (PsA) and undifferentiated arthritides.^45^ The estimated European annual incidence of JIA is 8.2 (95% CI, 7.5–9.0) per 100,000 with a significant variation between and across populations, occurring almost twice as often in girls (10.0/100,000) in comparison with boys (5.7/100,000).^44^ The pathogenesis of JIA is unknown, but the strong familial pattern of disease and human leukocyte antigen (HLA) associations indicate a complex genetic risk profile.^46^ There are also signals indicating that environmental risk factors, for example, a history of breastfeeding, may be at play.^47^

Uveitis occurs in up to a third of children with JIA across various countries,^34^ with most affected children developing a recurrent or chronic anterior uveitis.^48^ The median age of presentation is 5 years.^49^ Children with oligoarticular arthritis are at the highest risk (uveitis occurring in 16–25%) and those with polyarticular or systemic arthritis are at the lowest risk of JIAU (seen in 1–4%).^50^ Girls with early-onset oligoarticular arthritis who are seropositive for IgG antibodies recognizing nuclear antigens (antinuclear antibody (ANA) positive) are at the highest risk of developing chronic anterior uveitis.^34,49,51,52^ There are contrasting theories as to whether ANA positivity, gender or age at diagnosis confers the greatest risk. Recent studies indicate that the different genetic traits in JIA may confer differential uveitis risk in girls and boys.^53^ ANA positivity is also seen in cases of recurrent or chronic anterior uveitis in children without arthropathy. These children may have an atypical or attenuated form of JIAU.

The classical presentation of a delayed diagnosis of JIAU is the previously asymptomatic child who develops strabismus (unilateral disease) or evidence of poor vision (bilateral disease) with a ‘white eye’, band keratopathy, anterior chamber inflammation, posterior synechiae and cataract, with secondary disc and macular oedema.^2^ This is uncommon in modern practice, thanks to two developments: the wide adoption of national uveitis surveillance programmes, in which children with JIA are regularly checked for the onset of ocular inflammation,^43,54,55^ and the increasing use of systemic immunosuppressive therapies for children with JIA.^56–58^

Treatment for JIAU or ANA-positive chronic anterior uveitis (the latter thought to be a ‘forme fruste’ of JIAU) acts as a paradigm for the general management for noninfectious childhood uveitis. Inflammation which remains uncontrolled despite 3 months of topical corticosteroids is in most cases an appropriate indication for the use of a systemic disease-modifying steroid sparing agent.^34^ These agents may take up to 3 months to reach full efficacy, necessitating the use of temporizing oral steroids in cases of severe, sight-threatening levels of inflammation.

Methotrexate, an antimetabolite, is the first choice for chronic anterior uveitis, but can be associated with significant nausea, malaise or iatrogenic liver inflammation in a minority of children.^56^ Mycophenolate mofetil has been used for those children unable to tolerate methotrexate, and there is evidence of noninferiority in the management of most forms of uveitis when compared with methotrexate in adults.^59^ It is, however, a poorer therapeutic choice for JIA arthropathy.^34^ Insufficient response to these agents is an indication for a ‘step-up’ on the therapeutic ladder, with the introduction of a tumour necrosis factor inhibitor.

The use of adalimumab (an anti-TNFα agent) has improved disease control for adult and paediatric inflammatory eye disease,^60^ and following strong evidence of efficacy and safety in JIAU refractory to methotrexate,^60^ and in other uveitides,^61–63^ adalimumab has been commissioned in the United Kingdom for use in childhood-onset anterior and posterior uveitis. With regard to uveitis management, adalimumab has the advantage over other anti-TNFα agents such as etanercept and infliximab due to exhibiting greater disease control *versus* the former (in the form of fewer recurrences)^64–67^ and subcutaneous delivery rather than intravenous delivery for the latter.^64^ Infliximab has also been associated with greater rates of anaphylactic responses on administration due in part to the chimeric mouse–human nature of the molecule *versus* the fully human recombinant antibody of adalimumab. There is also evidence of effectiveness on switching to a second anti-TNFα in children with disease refractory to the first, specifically switching to adalimumab or to infliximab from etanercept.^64^

Newer agents targeted against inflammatory factors such as IL-6 (e.g. IL-6 monoclonal antibody, tocilizumab) and janus kinase (JAK; e.g. JAK-inhibitor baricitinib) have also emerged as possible therapeutic agents for childhood uveitis. Interleukin-6 has been postulated as a key mediator for inflammatory macular oedema, and tocilizumab appears to be of particular benefit in individuals with uveitic macular oedema.^68–70^ The janus-associated kinase pathways mediate cell responses to several inflammatory cytokines involved in uveitis including IL-2 and IL-6, and JAK inhibitors such as baricitinib and tofacitinib have been shown to control disease in individuals whose uveitis has failed to respond to other immunomodulators.^71^

Two of the JIA subtypes, PsA and ERA, are associated with HLA-B*27 positivity. HLA-B*27-positive acute anterior uveitis (AAU), presenting with a red painful eye, is one of the most common forms of adult uveitis.^72^ Many children with ERA or psoriatic JIAU present with a symptomatic uveitis, but the majority (57% in one population-based study)^43^ of these children present more insidiously. HLA-B*27 is also associated with spondyloarthropathy, reactive arthritis and inflammatory bowel disorders. There is growing evidence that HLA expression is altered in gastrointestinal (GI) disease and along with changes in the gut microbiome may suggest a mechanism of disease pathogenesis in these individuals^73^ and open avenues to new therapeutic options.

Although JIA is a disorder of childhood onset, activity often continues into adulthood, with an ongoing need for topical and systemic therapy and ocular surgery, and an ongoing negative impact on vision-related quality of life.^74,75^

### Sarcoidosis and NOD2-associated autoinflammatory disease (Blau syndrome)

Sarcoidosis is a chronic inflammatory disorder characterized by a granulomatous response, which particularly manifests in the lungs and lymphatic system. It is a rare disorder in adults, and even more so in childhood, with a cumulative annual incidence of 0.3–0.8 per 100,000 children.^76^ Sarcoidosis typically presents in older children, with a mean age at diagnosis of 11–13.^24^ The proposed pathogenesis of disease involves a ‘two-hit model’, in which genetic predisposition (‘first hit’) is combined with environmental exposure to a triggering environmental agent. Hypothesized second hit agents include foreign antigen, tuberculosis^77^ and inorganic material such as silicone.^78^

Almost a half of children diagnosed with sarcoidosis develop ocular disease (Box 1), which may in some cases predate the systemic features.

**Box 1.** International Workshop on Ocular Sarcoidosis (IWOS) criteria for the diagnosis of ocular sarcoidosis (OS).^79^

Other causes of granulomatous uveitis must be ruled out. In children this includes NOD2 syndrome, tuberculosis or other mycobacteria, immune deficiency disorders, eosinophilic granuloma, Crohn’s disease, tumours, drug-induced granulomatosis
Intraocular clinical signs suggestive of OS
Mutton-fat keratic precipitates (large and small) and/or iris nodules at the pupillary margin (Koeppe) or in stroma (Busacca) Trabecular meshwork nodules and/or tent-shaped peripheral anterior synechia Snowballs/string of pearls vitreous opacities Multiple chorioretinal peripheral lesions (active and atrophic) Nodular and/or segmental periphlebitis (±candle wax drippings) and/or macroaneurysm in an inflamed eye Optic disc nodule(s)/granuloma(s) and/or solitary choroidal nodule Bilaterality
Systemic investigation results in suspected OS
Bilateral hilar lymphadenopathy (BHL) on chest X-ray and/or CT scan Negative tuberculin test or interferon-gamma assays Elevated: serum ACE OR serum lysozyme OR bronchoalveolar lavage fluid CD4/CD8 ratio (>3.5) Abnormal accumulation of gallium-67 scintigraphy or 18F-fluorodeoxyglucose positron emission tomography imaging Lymphopenia Parenchymal lung changes
Diagnostic criteria
Definite OS: diagnosis supported by biopsy^a^ with compatible uveitis Presumed OS: diagnosis not supported by biopsy, but BHL present with two intraocular signs Probable OS: diagnosis not supported by biopsy and BHL absent, but three intraocular signs and two systemic investigations are present

ACE, angiotensin-converting enzyme; BHL, bilateral hilar lymphadenopathy; CT, computed tomography; IWOS, International Workshop on Ocular Sarcoidosis; OS, ocular sarcoidosis.

a Corticosteroids can mask granuloma after only a few days of treatment.

Methotrexate and mycophenolate mofetil are first-line agents for childhood ocular sarcoidosis, with no consistent evidence of superiority of either agent, but continued preference for the use of methotrexate due to its status as the most studies immunomodulatory for chronic ocular inflammation. Anti-TNFα agents are typically used for those refractory to their first-line treatment, although clinicians may first ‘switch’ nonresponding patients from methotrexate to mycophenolate in cases of nonresponse and vice versa.^76,80,81^

Edward Blau^82^ described a family affected by early-onset arthritis, dermatitis and uveitis, with granulomatous skin lesions consistent with sarcoidosis. Blau’s syndrome, however, lacks the pulmonary features which are present in the majority of paediatric sarcoidosis cases, and has an earlier median age at uveitis onset (5 years, with median onset of arthritis at 2 years old).^76^ Multiple genotypes for Blau syndrome exist, and it is possible that the many different mutations of NOD2 confer different risks of ocular involvement. Panuveitis with chronic multifocal chorioretinitis is the most common ocular finding, although a third of children have an isolated chronic anterior uveitis.

### Behçet’s disease

Behçet’s disease (BD) is a relapsing-remitting variable vessel vasculitis (i.e. affecting large and small veins and arteries), with a phenotype that differs depending on age at onset, gender, ethnicity and country of residence. Children appear to manifest ‘full-blown’ disease slower than adults (Box 2), leading to delays in diagnosis.^86,87^ The typical hallmark features of paediatric BD are painful, recurrent, oropharyngeal, perianal and genital ulceration (Box 2).^85,88^ In an echo of the later diagnosis of monogenic Blau syndrome in cases previously thought to be ‘early-onset sarcoid’, a monogenic mimic of early-onset paediatric BD, haploinsufficiency of TNFα induced protein 3 (TNFα-IP3), has been described.^89^

**Box 2.** Diagnostic criteria sets for BD.

Major diagnostic features GA: genital aphthosis (recurrent painful, sharply defined, round or ovaloid shallow ulceration, including perianal) OA: oral aphthosis OM: ocular manifestations Minor diagnostic features NM: neurologic manifestations (vascular or parenchymal involvement) SM: skin manifestations (necrotic folliculitis, acneiform lesions or erythema nodosum) VM: vascular manifestations (venous thrombosis, arterial thrombosis and arterial aneurysms)
Diagnostic criteria set for paediatric BD
Any three out of the six criteria^PEDBD^
Diagnostic criteria set for adult BD
⩾ 4 points (2 points for major, 1 point for minor and 1 point for a positive pathergy test (pustular response to blunt dermal needle prick) test if carried out in at least 90% of patients)^ICBD^ OR OA + 2 out of remaining criteria^ISG^

BD, Behçet’s disease; GA, genital aphthosis; ICBD, International Criteria for Behçet’s disease;^83^ ISG, International Study Group;^84^ NM, neurologic manifestations; OA, oral aphthosis; OM, ocular manifestations; PEDBD, paediatric Behçet’s disease; SM, skin manifestations;^85^ VM, vascular manifestations.

Ocular manifestations of BD also appear to be less common in childhood (45% of children with BD *versus* 70% in adult BD).^85^ Uveitis in BD has been described as ‘explosive’ with aggressive sight-threatening recurrences of inflammation and an associated vasculitis which can be necrotizing and obliterative, leading to ischaemic damage to the macular or optic nerve. The mobile sterile transient hypopyon seen in adult BD also occurs in paediatric disease, as do episcleritis and scleritis. Boys with BD are more likely to develop uveitis and more likely to have severe disease, sight-threatening retinal vasculitis.^88^

Treatment with anti-TNFα agents has been shown to be effective in BD, but there is significant heterogeneity in this disease population, and a significant number with disease refractory to anti-TNFαs. Azathioprine and cyclosporine A have been shown to be effective for the posterior uveitis seen in BD, as have biologic agents targeted against the IL-1 family of cytokines (such as anakinra and canakinumab) which are elevated in patients with BD.^17,90,91^

### Inflammatory bowel disease

Ulcerative colitis (UC) and CD are chronic granulomatous GI diseases with extraintestinal manifestations such as arthritis and uveitis, which can occur many years before the onset of GI disease. There is a lower prevalence of ocular involvement in children (0.6–1.8%) than in adults (2–6%).^23^ Affected children can develop uveitis, which is usually a painful bilateral anterior uveitis, although studies in which entire populations of children with inflammatory bowel disease (IBD) have been examined report a uveitis prevalence of 9% in UC and 1% in CD. Children can also develop retinal vasculitis, and vascular occlusion, retrobulbar neuritis and keratopathy. Children with CD are much more likely to present with uveitis than those with UC.^23,37^

### Tubulointerstitial nephritis and uveitis

Tubulointerstitial nephritis and uveitis (TINU), in which there is immune-mediated inflammation causing acute kidney injury, typically presents in older or mid-teenage children, with new-onset nonspecific symptoms including fever, weight loss, fatigue, nausea, anorexia, arthralgia and myalgia.^92,93^ As with many other immune-mediated disorders, such as Vogt–Koyanagi–Harada (VKH), girls are more likely to develop disease with a female-to-male ratio within TINU populations of 3:1.^94^ The uveitis can precede the TIN, but in most cases uveitis occurs with or follows the kidney injury, appearing within 6 months of TIN presentation.^94^ The majority of children develop a chronic anterior uveitis, although posterior involvement (retinal periphlebitis, haemorrhages, multifocal chorioretinitis) is also seen. As with other rare disorders, diagnosis can often be a challenge: tubulointerstitial nephritis also occurs in sarcoidosis, BD and IBD.

### Vogt–Koyanagi–Harada

Vogt–Koyanagi–Harada is an idiopathic multisystem granulomatous disease, in which a dysfunctional immune response is directed against melanin-associated antigens within the eye, inner ear, meninges, hair and skin, leading to acute-onset meningoencephalitis, dysacusia and tinnitus, and later vitiligo and poliosis.^95,96^ Vogt–Koyanagi–Harada patients overwhelmingly present in adult life; however, one group in Turkey describes 15% of VKH cases developing in childhood in a retrospective review of seven tertiary centres.^95^ Children, like adults with VKH, typically present with relatively acute onset bilateral exudative detachments, disc oedema and peripheral choroiditis, which can then progress to chronic refractory anterior uveitis. However, children are more likely to have sight-threatening disease than adults.^93^

### Multiple sclerosis

Between 0.1% and 1% of adults with intermediate uveitis (IU), particularly those with peripheral retinal vascular sheathing, go on to develop demyelinating disorders.^97^ A similar pattern has not been seen in intermediate uveitis of childhood onset, although there is a paucity of published research of sufficiently long follow-up to confirm the true later life prevalence in affected children.^98^ Much of intermediate uveitis in childhood is idiopathic pars planitis, a defined subtype of IU characterized by diffuse vitreous cells, peripheral retinal vasculitis, inferior vitreous inflammatory condensates (‘snowballs’), pars plana exudation (‘snowbanks’) and macular oedema. Pars planitis is not known to be associated with any systemic inflammatory disorders.

Multiple sclerosis has traditionally been known as an autoimmune disease, but there is emerging evidence of the role of inflammasomes (i.e. autoinflammatory mechanisms) in the pathogenesis of certain MS endophenotypes, particularly those of early onset and/or associated with genetic markers.^18^

### Cryopyrin-associated periodic syndromes

The growing number of very rare monogenic autoinflammatory disorders are typically characterized by episodes of fever and inflammation due to dysregulation of the proteins involved in innate immunity, such as IL-1β. Within this group sit the cryopyrin-associated periodic syndromes (CAPS) which comprise three diseases of worsening severity: familial cold autoinflammatory syndrome (FCAS), Muckle–Wells syndrome (MWS) and neonatal-onset multisystem inflammatory disease or chronic infantile neurologic cutaneous articular syndrome (NOMID/CINCA).^22^

Children with CAPS can develop chronic or ‘periodic’ symptoms, with fever, rash and musculoskeletal and neurological involvement such as seizures or hydrocephalus. Over two thirds of patients get ophthalmic involvement, ranging from conjunctivitis to optic atrophy to a chronic anterior uveitis or panuveitis.^99,100^

The wide heterogeneity of the CAPS phenotype is partly explained by the many different mutations of the inflammasome NLRP3 gene which have been described in CAPS patients.^100^ Another inflammasome protein, pyrin, is encoded by the MEditerranean FeVer (MEFV) gene, mutations in which cause Familial Mediterranean Fever (FMF). Familial Mediterranean Fever is the most common inherited autoinflammatory disease, but although it is more common than CAPS, ocular manifestations (such as uveitis) are seen less common.^17,101^ In addition to monogenic ‘periodic fever’ disorders, there are also polygenic and/or acquired syndromes.^102^

## Uveitis in the paediatric vasculitides

Vasculitis can be a coexisting disease seen with, or as part of, an autoinflammatory disorder (e.g. in BD). Retinal vasculitis may be a common end result of a number of inflammatory events, from raised tissue levels of IL-1, IL-6 or TNFα to tissue microdamage caused by ischaemia.^103^ Uveitis is a recognized feature of antineutrophil cytoplasmic antibody (ANCA)-associated vasculitis (e.g. granulomatosis with polyangiitis), vasculitis within connective tissue disease (e.g. systemic lupus erythematosus or scleroderma) or small or medium vessel vasculitis [e.g. IgA vasculitis/Henoch–Schönlein purpura and Kawasaki disease (KD)].^93^ In KD, a mild, bilateral uveitis in a child with red eyes and a nonspecific fever can be one of the earliest signs of disease, and early diagnosis of KD is of particular value as late diagnosis can result in secondary chronic coronary artery disease.^104^

## Managing childhood uveitis

Prompt diagnosis of uveitis affords the child the best chance of disease control before the development of inflammation-related complications. As many children with the most common form of disease, anterior uveitis, do not present with sudden onset of pain and redness, active surveillance for disease is needed to ensure case detection. This is particularly important for children known to be at risk. Those diagnosed with JIA are advised to undergo an eye examination within 6 weeks of diagnosis and to continue with 3- to 4-monthly examinations to a duration in line with national protocols in which children are stratified by JIA type, age at diagnosis and ANA status.^54^ As many children may have isolated idiopathic uveitis, or have uveitis as a presenting manifestation of a systemic inflammatory disease, there may be no useful indicator that the child is at risk until they present with established, sight-threatening disease.

The most common causes of sight loss in childhood uveitis are glaucoma, cataract and macular oedema.^2,55,105^ These occur as a sequelae of either uncontrolled inflammation or injudicious use of topical steroids.^9,106–108^ Thus, adequate control of ocular inflammation is key to good visual outcome for the majority of children. This often requires the use of powerful immunomodulators or immunosuppressive agents, in order to limit the dependence on the topical and systemic corticosteroids needed to control disease. The use of such systemic therapies necessitates the involvement of child health specialists and nurse specialists.

Almost one in six children and young people with uveitis develop severe visual impairment in one eye before adulthood.^105,109^ Although poor vision in only one eye often has a minimal impact on overall developmental and socioeconomic outcomes,^110^ uveitis is usually bilateral and often remains active into adulthood, putting the seeing eye at risk.^111^ Consequently, children who lose vision in one eye can then go on to lose vision in the other eye in adulthood, with a significant negative impact on their quality of life. In addition, children and the adults they become carry the burden of being diagnosed with a rare, often idiopathic disease of a chronic nature, which may necessitate treatment with immunosuppressive therapies which themselves carry the risk of short- and long-term morbidity.^75,112–115^ Multidisciplinary approaches (involving ophthalmologists, child health specialists, paediatric rheumatologists and where necessary psychosocial services) enable early diagnosis of associated conditions and holistic support for the child and family, which is key to good developmental outcomes.

## Summary

Childhood uveitis comprises a collection of heterogeneous ocular phenotypes which are associated with a diverse range of childhood immune and inflammatory mediated disorders which are genetic and/or acquired. The majority of disease is idiopathic. The diagnosis of most of the associated disorders is reliant on clinical features rather than serological or genetic investigations, and no one ocular phenotype fits exactly with any particular systemic disorder, necessitating detailed medical history taking and systemic examination. Adequate control of inflammation is key to good visual outcomes, and multidisciplinary care is key to broader health outcomes.
